# High Voltage Electric Fields Have Potential to Create New Physical Pest Control Systems

**DOI:** 10.3390/insects11070447

**Published:** 2020-07-15

**Authors:** Shin-ichi Kusakari, Kiyotsugu Okada, Manabu Shibao, Hideyoshi Toyoda

**Affiliations:** 1 Research Association of Electric Field Screen Supporters, Nara 631-8505, Japan; kusakari@mbox.kannousuiken-osaka.or.jp; 2 Agricultural, Food and Environmental Sciences Research Center of Osaka Prefecture, Osaka 583-0862, Japan; okada@mbox.kannousuiken-osaka.or.jp (K.O.); shibao@mbox.kannousuiken-osaka.or.jp (M.S.)

**Keywords:** physical control, electric field screen, discharge-mediated positive electrification, electric field avoidance, insect-repelling function, insect-capturing function

## Abstract

An electric field is the space surrounding an electric charge, within which it is capable of exerting a perceptible force on another electric charge. Especially under high voltage, electric fields induce various electrostatic phenomena, some of which could be utilized to provide remarkable pest control measures. The main focus of the present study was to introduce an attractive force generated by a surface charge on an insulated electrified conductor, which was successfully used to construct an electric field screen that prevented airborne nuisances (spores, flying insects, pollen, and fine smoke) from entering the interiors of various facilities. Another focus was the disinclination of insects to enter the electric field, thus, giving the electric field screen the ability to repel insects. Charges accumulated on the surfaces of non-insulated conductors are mobile through discharge, based on their potential difference. Such arc discharge was strong enough to destroy insects that were exposed to it. Some precedent illustrative examples are cited to explain the principles of attraction, dielectrophoretic movement of spores, and discharge-mediated positive electrification of insects, and to discuss how electric fields are generated and used in electric field-based pest control strategies.

## 1. Introduction

Pesticides have been the dominant method used for control of all classes of pests (pathogens, insects, and weeds). However, the overuse of pesticides has led to the rapid evolution of pesticide-resistant pests. Given the pesticide-resistance problem and consistent public concern to reduce overall pesticide use, pesticide-alternative strategies are urgently required. The protection of crop plants from infection and/or attack by pathogens and pests via safe and environmentally benign methods has been a long-standing goal. Much effort has focused on developing biological and chemical methods to achieve this, including the production of resistant crop plants using conventional and new biotechnological techniques, biocontrol of pathogens and pests using antibacterial, antifungal, and entomopathogenic microorganisms, and the screening of biologically synthesized compounds that inhibit growth of the pathogens [1,2]. Despite much interesting work, there has been little practical progress because the protective effects are easily destroyed, and because of problems with agent preparation, limited targets for application, and high costs. The principal problems facing practical implementation are the application of individual methods for pathogen and pest control at scales larger than the test experiments, and variable environmental conditions. These trials have taught us that the aforementioned techniques were, in essence, supplementary measures for a limited range of targets under particular conditions. We must reflect on the lack of reliable basic methods that can be combined to form an appropriate approach [1].

Once a realistic and reasonable research objective can be settled, it would be possible to advance our steps steadily through a cyclical repetition of creation and destruction of a working hypothesis formulated from the reproducible experimental results and eventually, to propose new conceptions for an electric field screen. An electric field screen is an air-shielding apparatus devised on the basis of principles and techniques of applied electrostatic engineering and was first introduced in a famous American specialist journal ‘Phytopathology’ in 2006 [3]. At the outset, the apparatus was put to the public as a new device to capture airborne conidia (spores) of phytopathogenic fungi during crop cultivation in a greenhouse. As the research progressed, the targets of the screen expanded from fungal spores to flying insect pests, pollen grains that cause pollenosis, and fine particles of tobacco smoke, as a result of optimizing the structure of the electric field screen and its capture capabilities. Thus, advances in electric field screen technology have allowed broader applications of the device, from agricultural fields, e.g., crop production, processing, and storage, to environmental and public health science fields [4].

A number of physical methods, especially exploiting electrostatic phenomena, can generate physical forces sufficiently strong to catch airborne fungal spores or small flying insects that may pass through the conventional insect netting used to protect greenhouse crop plants. If an effective force could be generated, such an electrostatic approach would be an extremely promising tool to provide a spore-free and pest-free space for crop plants in a greenhouse environment [5]. In this review, we describe the development of different types of electric field screens, including unique structures and electrostatic mechanisms for capturing insects and pathogens, focusing on the formation of a non-uniform static electric field. This paper provides diagrams and photographs, and in addition, videos are included to aid the reader in understanding how interesting the electrostatic world can be. Furthermore, we describe the nature of insects to avoid entering a static electric field, which confers an insect-repelling function on the screen and discharge-mediated electrostatic devices to instantaneously dismember the insects that enters the electric field.

Physical methods could provide an alternative means of managing the pests, since they would be compatible with other components of integrated pest management, have little impact on the environment, and reduce pesticide use, thus, slowing the development of pesticide resistance. In conclusion, the present work demonstrates that the basic electrostatic knowledge brought about a physical tool contributable to social benefits. Thus, the concepts and technologies originating in university laboratory are made readily available to the public in an effort to fulfill scientists′ mission of contributing to society by helping to solve real-world problems and move science forward.

The purpose of the present review work is to introduce some parts of the interesting phenomena of high voltage electric fields, which anyone can produce easily with a simple voltage generator, especially in terms of physical control of insect pests. From this point of view, previous works of this field could be substantial precedents for subsuming electric field-based techniques into the existing theory of pest management strategy. More importantly, touching the real phenomena of the electric field by the readers could be a trigger or chance to introduce a new approach to pest control or to improve the apparatus in response to special needs of insect pest problems. These challenging approaches are essential to clarify the total picture of what could happen to the insects in the electric field and how we should utilize these phenomena for constructing a new system of pest control approach. This review paper was prepared to make a first step for this purpose.

## 2. Generation of Electric Fields

**Figure 1:**
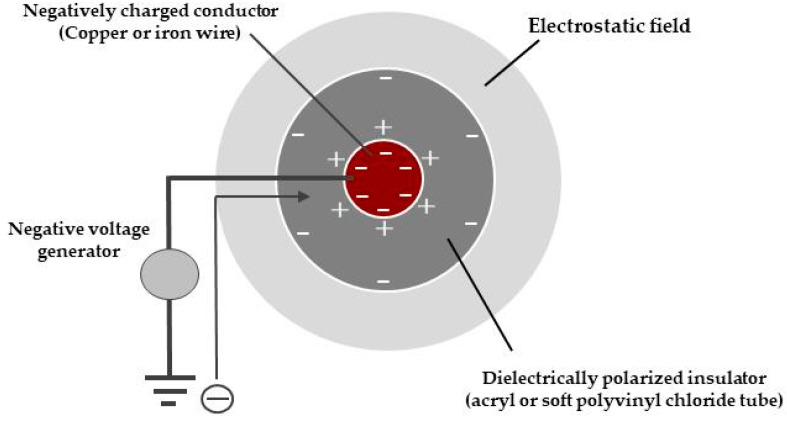
Schematic representation of the electrostatic field produced by a negatively charged insulator, covering a negatively charged conductor.

Electric field is defined as the space surrounding an electric charge, within which it is capable of exerting a perceptible force on another electric charge [6]. The electric field can be generated in the space surrounding the insulated conducting wire (metals such as copper and iron); the wire is electrified by connecting a negative voltage generator that is earthed (Figure 1).

A negative voltage generator draws negative electricity, free electrons, from the ground (an infinite source or sink of electrons—in this case, a source) and supplies electrons to the conducting wire [7]. Negative electricity accumulates on the surface of the wire conductor. In turn, negative charges are induced on the outer surface of the insulated coating of the wire, thus, negatively electrifying the insulator via dielectric polarization [8].

The amount of electricity required for electrification is proportional to the applied voltage of the voltage generator. As the applied voltage increases, the amount of electricity increases. The applied voltage corresponds to the potential difference with respect to earth ground. A larger potential difference creates more remarkable electrostatic phenomena as a result of the larger attractive or repulsive force generated. This allows for the capture of insect pests.

The application of negative voltage to an insulated conductor allows negative charge to accumulate on the conductor surface, which, in turn, produces an electric field in the surrounding space. This field induces surface charges in the insulating coating of the conductor. In our example, in which the insulator is an acrylic cylinder, opposite charges are created on the inner and outer surfaces of the cylinder. The outer surface charge of the cylinder (a monopole) produces an electrostatic field in the surrounding space (Figure 1), i.e., there is no current flow (no flow of electricity) in this case, only space charge. The field intensity is strongest in the region closest to the charged/electrified body, in our case, the insulator. The gradient of the electric field intensity is used to capture objects that enter into the field.

**Figure 2:**
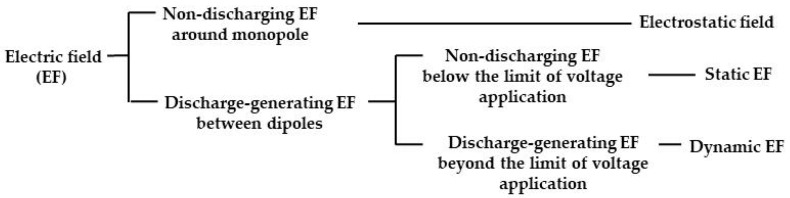
Classification of electric fields formed in the electric field screen.

Figure 2 shows various electric field configurations. An electrostatic field is formed in the surrounding space of a charged, insulated conductor used as a single pole. In this field, the electricity that accumulates on the charged conductor is not released (namely, not discharged) in the field. In contrast, the electric field formed between oppositely charged poles may result in an electrical discharge if the applied voltage exceeds a certain limit, regardless of whether the conductor is insulated or not. This type of electric field is distinguished by the presence or absence of a discharge. Hereafter, the non-discharging electric field is referred to as a static electric field, and the discharge-generating electric field as a dynamic electric field (or merely, electric field).

## 3. Discharge-Mediated Positive Electrification of Insect in a Static Electric Field

Acrylic resin has a pronounced insulative nature, such that a high voltage can be applied to the conductor insulated with this resin. At the same time, this resin is highly difficult to process. This problem was a cause of concern, especially in the development of new types of electric field screens and their future practicality in real-world applications. To solve this problem, we turned to polyvinyl chloride resin, commonly used to insulate metal materials. However, as we had no equipment in our laboratory capable of producing this resin or coating metals with this resin, we had to substitute commercially available soft polyvinyl chloride tube (Toalon^®^ tube, Kokugo, Tokyo, Japan). The most serious drawback of using soft polyvinyl chloride tube is that its volume resistivity is considerably low, thus, limiting the ability to charge the material. Unexpectedly, this limitation was the motivating force to bring about a new method to create the necessary capture capabilities with a lower voltage application [5].

The major factors to be considered in the design of the electric field screen are: (1) the quality of the insulation material, (2) pole distance, (3) range of applied voltage, and (4) the presence or absence of discharge (especially, arc discharge). All of these factors are closely related. For example, for the same applied voltage, if the pole distance is shortened, discharge occurs more easily between the opposite poles. Considering this situation, we modified several of the screen parameters, such as the applied voltage, while allowing some of the factors to remain fixed. Specifically, the fixed parameters included the use of soft polyvinyl chloride tube to insulate the conductors and a 5 mm separation distance (interval) between adjacent insulated conductors used to create the screen. In the experiment, the insulated conductor wire was fabricated by passing a metal wire (copper or iron wire) through a soft polyvinyl chloride tube. The wires were then arranged in a parallel configuration, with a constant separation interval of 5 mm as a common skeletal structure for subsequent electric field screen designs.

**Figure 3:**
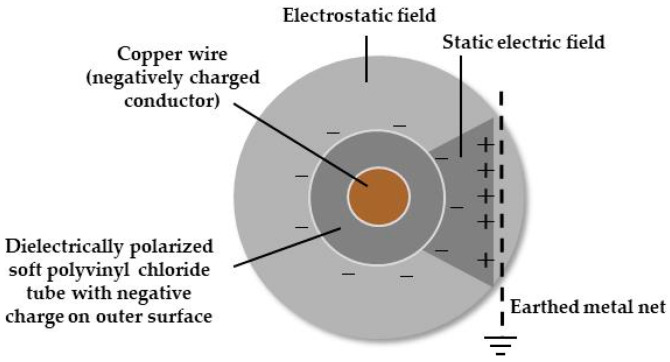
Formation of a static electric field inside the electrostatic field, formed by a single-charged monopolar electric field screen.

During the electric field screen research, we devised a new method by which a dipolar static electric field could be generated via single charging (Figure 3). The electric field screen integrating this field exhibited a revolutionary power for capturing insect pests.

An insulated conductor wire charged with a negative voltage generator causes dielectric polarization within the insulating coating, thus, creating a negatively charged insulator surface. The charged surface of the insulator produces an electrostatic field in the surrounding space. Thus far, this is the same as the acrylic cylinder examples described earlier. The difference for the single-charged dipolar electric field screen design is that, at this point, we placed a grounded metal net inside the electrostatic field produced by the insulated conducting wire (Figure 3). The grounded (earth ground) metal net facing the charged insulated conductor becomes positively charged as a result of electrostatic induction [8]. Remember, the insulation surface of the insulated conducting wire has a negative charge. Thus, given that earth ground is a source or sink of electrons, the free electrons in the metal net are pushed away to earth ground by the negative insulator surface, as like charges repel. The deficiency of electrons in the metal net leaves the net with a positive charge. The opposite charges of the insulated conductor wire (negatively charged) and the earthed net (positively charged) create a dipole, forming an electric field in the space between them. If we select a voltage range that causes no discharge from the charged insulated conductor wire, the poles create a static electric field between them. Thus, we can create a positive pole (earthed metal net) without using a positive voltage generator. This is our single-charged dipolar electrification system [9].

In the assay of insect capturing, a test insect is collected with an insect aspirator pipe and is then blown into the electrostatic or static electric field. Here, we examined the capture rate as a function of the applied voltage. Successful capture was judged by blowing; the insect was deemed as being captured completely if the insect remained held against the insulated conductor wire as it was blown upon at 7 m/min for 10 s. In the electrostatic field, the insect is attracted to the charged insulated conductor wire by dielectrophoresis [10]. However, the force generated is not sufficiently strong for insect capture. Indeed, the captured insect struggled desperately to free itself of the attractive force of the insulated conductor wire and finally, escaped. The power struggle is less for larger (size) insects. Therefore, the ability to capture the larger insects becomes more difficult. In this case, it is impossible to completely capture the insect, even if the applied voltage is enhanced. In contrast, in the static electric field created by the single-charged dipolar electric field screen, a strong attractive force is generated between the charged insulated conductor wire and the insect, which was positively charged by the release of free electrons. The insect cannot escape, despite its struggle to get free. Regardless of insect size, this type of screen captured the insect every time.

**Figure 4:**
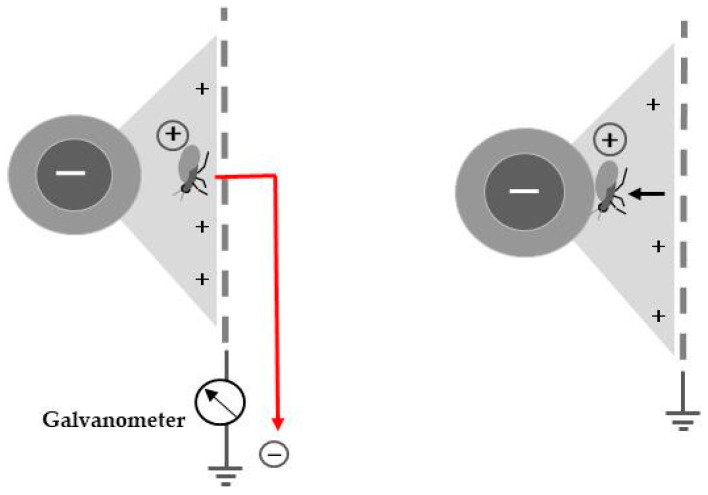
Schematic representation of insect capturing by discharge-mediated positive electrification of an insect in the static electric field.

A major characteristic of the static electric field is the negative charge of the insulated conductor wire. The negative charge creates a strong repulsive force to other negative charges (electrons) in the electric field, pushing them toward the ground via the metal net (Figure 4). By this mechanism, any conductor that enters this field is deprived of its free electrons (negative electricity) and becomes electrified positively (positively charged). This phenomenon is called discharge-mediated positive electrification of the conductor [5].

The theme of this section is the insect itself and how it responds to the static electric field upon entering. Most insects possess a solid cuticle layer, which is the outer protective layer that covers the body of many invertebrates. This layer is known to be conductive; thus, an insect that enters the static electric field is deprived of its free electrons in the cuticle layer and becomes positively charged. This implies that discharge-mediated positive electrification can be induced in the actual insect, as the free electrons of the cuticle layer move to earth ground. A galvanometer (a kind of ammeter used for detecting electric current) can be integrated into the ground circuit to detect this type of electron movement. Positively electrified insects are attracted to the central insulated conductor wire (Figure 4). This force is so strong that the captured insect cannot escape from this trap [11,12]. This capturing mechanism is applicable to almost all insects, as they tend to possess a conductive cuticle layer.

**Figure 5:**
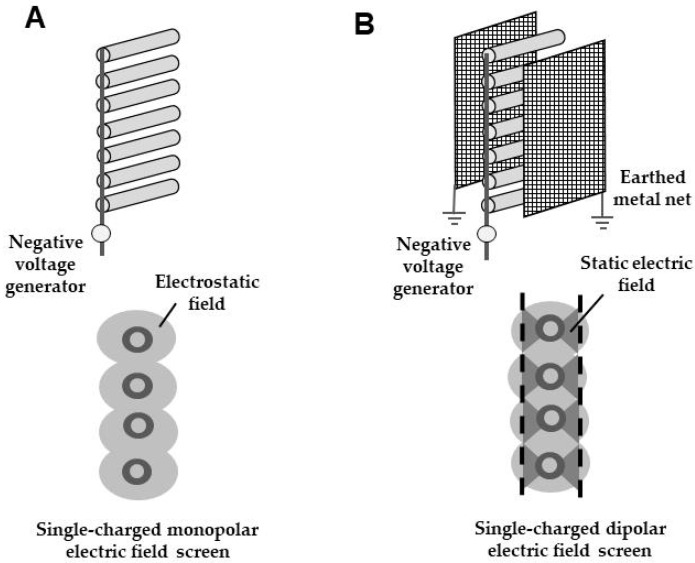
Comparative representation of single-charged monopolar (**A**) and dipolar (**B**) electric field screens.

As shown in Figure 3, the static electric field is formed inside the electrostatic field. If we place another earthed net on the opposite side of the insulated conductor wire, the static electric field forms in a manner consistent with bilateral symmetry. Accordingly, if we arrange the insulated conductor wires such that the upper and lower ends of the two static electric fields contact each other, a new electrostatic barrier of static electric fields is constructed. A single-charged monopolar electric field screen is constructed by insulating conductor wires with a soft polyvinyl chloride tube, arranging them in parallel at constant intervals and linking them to each other and to a voltage generator (Figure 5A) [3]. A single-charged dipolar electric field screen is formed simply by placing an earthed metal net inside the electrostatic field that was produced by a single-charged monopolar screen (Figure 5B). However, there is a remarkable difference in their insect-capturing capabilities.

Four kinds of greenhouse pests were included in the tests: whitefly (*Bemisia tabaci*), Western flower thrips (*Frankliniella occidentalis*), green peach aphid (*Myzus persicae*), and tomato leaf-minor fly (*Liriomyza sativae*) [13]. All of these pests can pass through a conventional insect-proof net (mesh size, about 5 mm). The sizes of the test insects increased in the order presented above, with tomato leaf-minor fly being the largest among them. The earthed metal net of the electric field screen is a stainless steel net with the same mesh size (about 5 mm). Larger pests that could not pass through this size of mesh were not tested, as they were prevented from entering by the net, without the need for an electric field screen.

At real farming sites, small insect pests pose a serious threat to crops. Three of the pests listed above cause particularly severe viral diseases, in addition to damage by pest attack. Additionally, whitefly, Western flower thrips, and green peach aphid carry the tomato yellow leaf curl virus (TYLCV), tomato spotted wilt tospovirus (TSWV), and the cucumber mosaic virus (CMV), respectively. All pests have already acquired resistance against the major insecticides currently available [13].

We conducted the insect-capturing assay using an electric field screen-installed greenhouse [14]. The results are summarized below: (1) larger voltages are required to capture larger pests, (2) in all pests, higher voltages produce stronger force for capture, (3) charging at 4.2 kV was sufficient for capturing all of the pests tested, and (4) captured insects were held tightly against being blown at 7 m/s. From these results, the electric field screens installed in the greenhouse were charged at 4.2 kV for field experiments. As a practical note, ordinary greenhouses possess a system to close the windows automatically or manually when the outside wind velocity reaches 3–5 m/s.

## 4. Insect-Repelling Function Based on Insects’ Habit to Avoid a Static Electric Field

**Figure 6:**
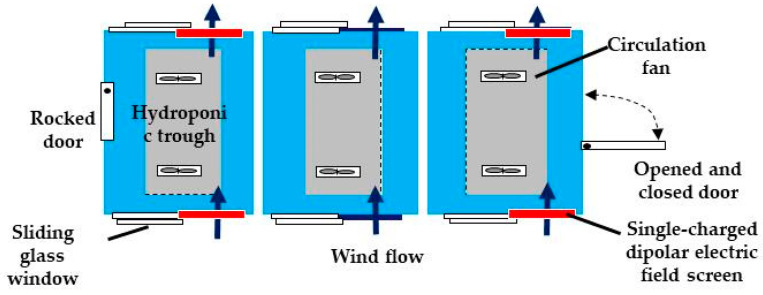
Experimental setup for an insect exclusion assay in three divided rooms of a greenhouse.

In this particular experiment, the greenhouse was divided into three rooms using partitions (Figure 6). The purpose of this experiment was to examine the entry of insect pests into the divided rooms and the trapping of these pests with single-charged dipolar electric field screens installed in the windows of both end rooms [15]. The number of pests that entered the three rooms was determined by counting the number of pests trapped by the yellow- and blue-colored adhesive plates hung in each room. Notably, many whiteflies and tomato leaf-minor flies were trapped by the yellow plates in this experiment.

The results obtained clearly show the exclusion of pests from the greenhouse by the electric field screen [15]. First, the non-guarded room (center room) received an average of 1000 entries of pests over the two-week experimental period. In contrast, no pests were observed on the adhesive plates of screen-installed, door-locked rooms.

In one side room that allowed entry/exit of workers during the experiments, a few pests entered the interior from the door entrance. Nevertheless, the rate of pest exclusion was more than 92%, indicating that the electric field screens successfully prevented pest entry from the windows [15]. An interesting result was the number of pests captured by the screens installed in the two side rooms. In fact, numerous pests invaded the central room; therefore, it is reasonable to assume that similar numbers of pests attempted to invade both side rooms of the same greenhouse. Thus, initially, we expected to find that the electric field screens of these side rooms had trapped the same number of pests acquired in the central room. However, the real result was that fewer insects had been trapped. This suggests that the electric field screen actually repels the pests due to the low numbers captured in the end rooms [15].

To prove this repellence function, an acrylic cylinder with an axial fan was constructed to examine the behavior of the pests placed inside. The cylinder was in contact with the grounded metal net of the screen to observe the insects reaching the net. Additionally, insects were blown from the back when they reached the net. Under non-blowing conditions, all of the pests that reached the net stopped and entered their antennae or legs inside the space of the static electric field of the screen, similar to a ‘searching’ behavior of the inside [15]. The pests were deterred from entering the static electric field of the screen. On the other hand, when the pests were blown from the back, almost all pests clung to the net string and took a posture to avoid the wind. However, some pests lost their balance and were forcibly pushed inside the screen by the wind. These pests were captured by the strong force of the electric field. These results are important as they confirm that all pests reaching the earthed net of the electric field screen attempted to avoid the static electric field (i.e., they were repelled by the screen), and had to be forcibly pushed inside the screen by the wind. This explanation is in good agreement with the demonstrative experimental results described in the previous section [15].

**Figure 7:**
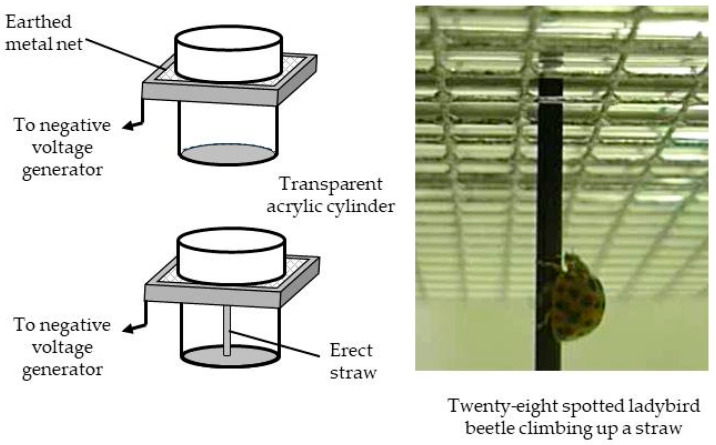
Apparatus for examining avoidance behaviors by the insects and a twenty-eight spotted ladybird beetle (*Epilachna vigintioctopunctata*) climbing up a straw.

A simple experimental instrument was constructed to examine the avoidance behavior of insects toward the static electric field of the electric field screen (Figure 7). Transparent acrylic cylinders were placed on and under the electric field screen, and test insects were put on the bottom of the lower cylinder, as the insects tended to climb upward. In fact, some insects flew up to the screen net, whereas others climbed up the wall or an erected pole/straw (e.g., ladybird beetles). With the non-charged screen, all insects passed through the screen. In contrast, with the charged screen, the insects exhibited avoidance behavior at the outer surface of the earthed net and did not continue with their upward direction of movement. This again demonstrates the avoidance of insects in that the transparent cylinders allowed for a visual account of this behavior.

The results of the insect avoidance assay indicated that all insects tested exhibited avoidance behavior with respect to the static electric field. The insects tested covered a total of 17 orders, 42 families, 45 genera, and 82 species [16]. From these results, we concluded that all insects are deterred by the static electric field of the electric field screen.

The results obtained here changed our way of thinking of how we should construct the electric field screen and what magnitudes of voltage should be applied to the screen. The research usually concentrated on enhancement of the ability of the screen to capture insects, namely, the direction of higher voltage application. Eventually, 4.2 kV charging was necessary for complete capturing of the insects. The basic requirement of the electric field screen was to create a pest-free space in the greenhouse. At this point, however, the screen was not necessary to exert insect-capturing function due to its prominent ability to repel almost all species of insects that reached the screen net. Charging of much lower voltages (0.8–1.2 kV) was high enough to deter the insects from entering the electric field screen. Technically, higher voltage application necessitates higher levels of protective measures to suppress an electric leakage at the joints of electrical wiring in the screen, and therefore, being attended with more high technical difficulty and larger costs. Contrarily, the insect-repelling function made it possible to construct the screen more easily and cheaper.

## 5. Practical Implementation of Single-Charged Dipolar Electric Field Screen

**Figure 8:**
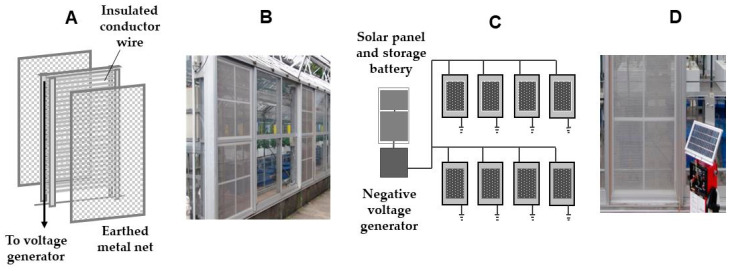
Installing of single-charged dipolar electric field screens to lateral windows (**A**,**B**) of the greenhouse and an electric circuit to link the screens (**C**) to a voltage generator (**D**).

The electric field screen first put to practical use was the single-charged dipolar type produced by our laboratory (Faculty of Agriculture, Kindai University) (Figure 8A) [5]. As shown in the photograph below, the screens were installed on lateral windows of the experimental greenhouse (Figure 8B). Four screens on each side of the greenhouse—eight screens in total—were installed. All screens were linked to each other and driven by one voltage generator (Figure 8C). The voltage generator was operated by a storage battery powered by a solar panel. The electric power consumption by the voltage generator was small, such that an ordinary lithium storage battery would be applicable (of course, normal alternating current (AC) domestic power sources could also be applied). A commercially available voltage generator combined with the storage battery with a small solar panel was used for the experiment (Figure 8D).

In the actual greenhouse experiment, the greenhouse was separated into two rooms by a partition. In one of the rooms, all of the lateral windows were furnished with single-charged dipolar electric field screens. The screens were negatively charged at 4.2 kV, as described in the previous section. In the second room, there were no screens installed. We examined the entry of insect pests into both rooms. The period of each experiment was two weeks, and six experiments were conducted in total. The ventilating fan was also covered with an electric field screen box to prevent pests from invading via the opening left by the fan, especially when the fan stopped.

Over the course of the experiment, there were no occurrences of green peach aphid or Western flower thrips. However, moderate to severe whitefly and tomato leaf-minor fly invasions were evident over the same time period. As expected, both pests were completely prevented from getting into the screen-installed room in all series of experiments. In these demonstrative experiments, the practicality of the electric field screen was clearly proven. The severity of the pest invasion was clear from the number of pests trapped by the yellow-colored adhesive plates hung inside the room that was not furnished with an electric field screen. That an intense invasion occurred in one of the half rooms of the same greenhouse indicates that a similar invasion would have been likely in the other half room if preventive measures had not been put into place [4,5]. Our results demonstrate the effectiveness of electric field screens for pest control.

## 6. Dismemberment of Insect in a Discharge-Generating Electric Field (Dynamic Electric Field)

Discharge is defined as electric current generation between opposite poles as a result of the dielectric breakdown of gases in the electric field by the potential difference between the poles [17,18]. The corona discharge phenomenon dealt with in this chapter covers continuous discharge caused by an uneven electric field generated around a pointed tip of an electrode (needle pole). The glow around the pointed tip is called the corona.

**Figure 9:**
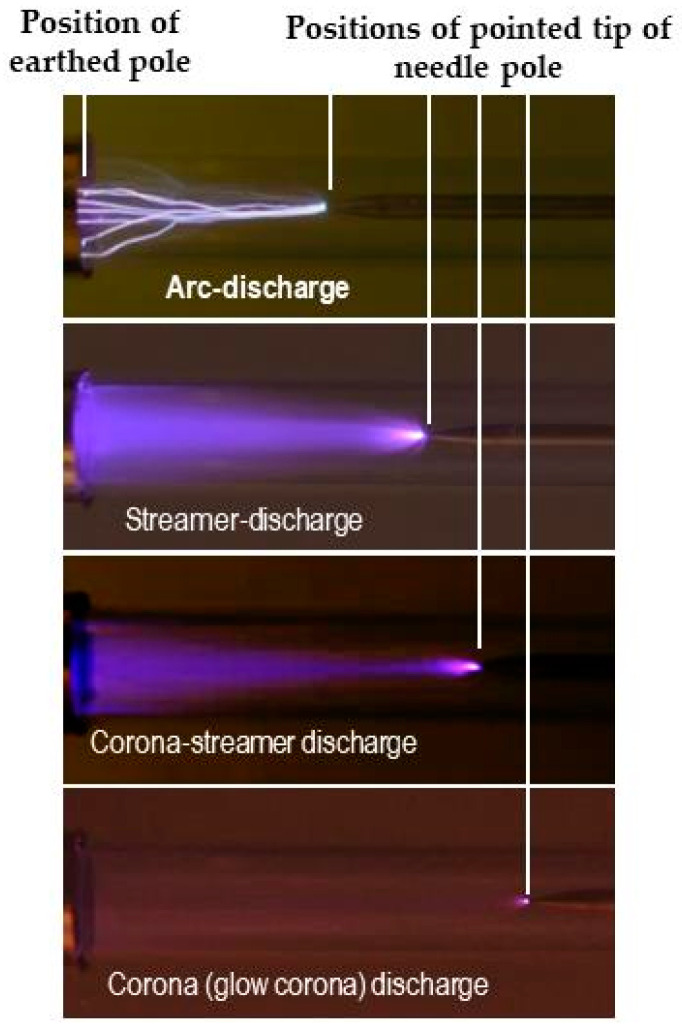
Different types of discharge caused by the dynamic electric field between a charged needle pole and earthed metal ring.

The state of the corona depends on the polarity of the needle pole (a distinction between positive- and negative-charging), the potential difference between poles, and the pole distance. In the case that the needle pole is positively charged, a corona discharge (positive corona discharge) can change from a glow discharge (corona discharge surrounding the tip portion) to a streamer discharge via a brush-like discharge by increasing the applied voltage and/or decreasing the distance between the poles. The discharge finally breaks down with an arc discharge between the two poles (Figure 9) [19]. If the needle pole is negatively charged, the corona is induced at lower voltages than the positive needle pole. Although glow corona with a short streamer discharge is generated, it does not become larger and is followed by a complete breakdown in the form of an arc discharge [20].

**Figure 10:**
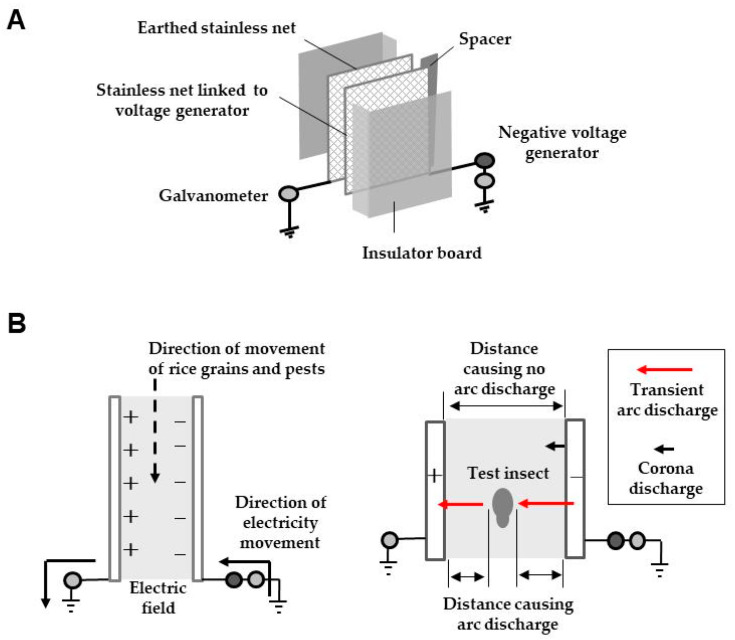
Schematic representation of an arc discharge-generating screen (**A**) and insect-mediated transient arc discharge generation (**B**).

The arc discharge-generating screen consists of two stainless steel nets [21]. One net is linked to a negative voltage generator and the other to a grounded line. The insulator board is attached to the outer surfaces of stainless steel nets to prevent grain (mainly rice grain) from falling through the net (Figure 10A). This screen is used to irradiate pests nesting in the grains directly (such as rice weevil, red flour beetle, Azuki bean weevil, and cigarette beetle) [22], with a transient arc discharge. This way, only the pests are dismembered. The screen structure is simple, but exhibits excellent functionality.

The principle is also simple. An electric field is formed between oppositely charged metal nets. One of the nets is negatively charged (negative voltage applied) to create the corona discharge. The glow corona is detected at the convex surfaces of the net (making for a shorter pole distance), which act as needle poles. Over the limit of voltage for corona discharge, arc discharge (mechanical discharge) occurs along portions of the net. This screen is an arc-discharge-generating type; however, it does not necessarily require mechanical arc discharge. When the insects enter the electric field between the nets, at any location, the insects effectively become intermediate poles. As such, these insects are subjected to an arc discharge from the negatively charged net due to their conductive cuticle outer layer.

Eventually, the electricity is transferred to the insect and then, to the earthed net via a two-step arc discharge (Figure 10B). This type of discharge is referred to as insect-mediated arc discharge [21]. This discharge is transient, but sufficiently strong for complete dismemberment of the insects, leaving no trace. If a higher voltage is applied, the rate of dismembered insect production increases. Even when the same voltage is applied, if the net area is enlarged, the rate of pulverized insects increases, because a larger amount of electricity accumulates and is then, released to the insect all at once.

## 7. Conclusions

The purpose of the present study was to introduce the potential abilities of high voltage electric fields to workers in the pest control research field. The most notable phenomenon regarding electric fields was the strong attractive force generated in non-discharging electric fields such as electrostatic and static electric fields. This attractive force formed the basis of the electric field screen function. The electric field screen was constructed as an air-shielding apparatus devised by harnessing the nature of the electric field. This apparatus was used to prevent airborne spores of pathogens, flying insect pests, pollen grains that cause pollenosis, and fine smoke particles from entering the inside of various facilities. The electric field screen is applicable to facilities such as domestic housing, hospitals and school buildings, greenhouses for crop production, warehouses and processing plants for post-harvest crops, and animal husbandry facilities. A wide variety of electric field screen structures can be customized to prevent the entry of biotic and abiotic environmental nuisances. All screens have a simple, common structure; therefore, the cost of production is relatively low. A single-charged, dipolar type of electric field screen is now commercially available because the conductors, which are the crucial components of the electric field screen, are coated with polyvinyl chloride resin to prolong screen operation in outdoor environments with minimal deterioration. This type of electric field screen is built and sold by a joint-stock company (Sonoda Seisakusho Co. Ltd., Osaka, Japan), which undertakes the design, installation, inspection, and maintenance of the electric field screens. Another application of attractive force was in the production of an electrostatic insect sweeper [23], which is useful for directly trapping insect pests such as whiteflies and aphids that frequently rest on leaf surfaces, and an electrostatic flying insect catcher (electrostatic racket) [24], which is an apparatus that is used to capture flying pests directly. Such equipment is carried by greenhouse attendants during ordinary plant care checks.

Another prominent feature of electric fields is their role in atmospheric events mediated through coronas, streamers, and arc discharge in dynamic electric fields. Using non-insulated conductors, discharge-generating electric field screens were constructed using the same method and configuration. The corona discharge-generating electric field screen produced negative ions to bind with fine particles of tobacco blown into the electric field, by which fine particles of tobacco smoke became trapped by the positively charged earthed metal net of the screen [25]. The electrostatic fungus-eradicating probe was a device that created a corona discharge around a negatively charged needle pole, from which a plasma stream was generated by the electric field between the needle pole and fungal pustules on the leaf surfaces of grounded plants [26]. The streamer discharge-generating screen produced ozone from oxygen molecules in the electric field and the ozone-containing air was bubbled into water to sterilize pathogens in water [19]. In spite of these active approaches, we have not, to date, succeeded in our efforts to apply these phenomena to pest control. Direct exposure of insects to arc discharge was the only method that was successful for pest control, by which the insect in the electric field was destroyed instantaneously as a result of the strong impact of the arc discharge [21].

The fact that insects avoid entering static electric fields indicates that single-charged electric field screens possess an insect-repelling function as well as insect-capturing ability [9]. The insect-repelling function is of primary importance for creating pest-free spaces inside facilities [16]. Interestingly, the insects had never before experienced such high voltage electric fields as those used in the experiment. The insects were strongly deterred from entering the electric field. Recent analysis revealed that some insects could perceive the existence of very weak electric fields [26], suggesting that insects may be good subjects for analyzing electric field biosensing abilities [27]. Thus, further accumulation of experimental data analyzing the behavior or responses of insects to electric fields will contribute to advancing electric field-based pest management approaches.

Our special perspective of future research ranges over many topics. The first is an application of the electric field screen to an unmanned aerial vehicle for remote-controlled monitoring of insects, especially ubiquitous flies transmitting plant and human pathogens over postharvest crops in the facilities of a food supply chain. For this purpose, the replacement of heavy conductor metal wires passed through an insulator tube with proper light materials was essential for considerable weight reduction of the screen. Coating of the inner surface of the insulator tube with conductive paste may be a potential alternative. The use of water in an insulative tube of the screen is a possible alternative due to its considerably higher electric conductivity. Interestingly, various water-soluble colorings provide the screen with a luminous body, which may be useful to analyze photoselectivity by insects. The combination of insulated and non-insulated conductive fibers may make it possible to weave them into a plastic sheet of the electric field screen. Discharge-generating techniques may provide a new tool to detect and dismember the insects nesting in dried grain under the inspection of postharvest crops, or to selectively kill some kinds of flies emerging from underground pupae, which may be useful for an organic farming system. These are only several instances among many possible applications of electric field-based pest control measures. Hopefully, this paper can be an opening to gather valuable research works in this new field.

## References

[1] Toyoda H., Matsuda Y., Nonomura T., Kakutani K., Takikawa Y., Toyoda H. (2015). Phytoprotection Science and Technology: Comprehensive Approaches to Crop Protection.

[2] Toyoda H., Matsuda Y., Nonomura T., Ouchi S. (2000). Molecular approaches to chemical and biological control of bacterial wilt pathogen of tomato. Plant Diseases and Their Control.

[3] Matsuda Y., Ikeda H., Moriura N., Tanaka N., Shimizu K., Oichi W., Nonomura T., Kakutani K., Kusakari S.-I., Higashi K. (2006). A New Spore Precipitator with Polarized Dielectric Insulators for Physical Control of Tomato Powdery Mildew. Phytopathology.

[4] Toyoda H., Matsuda Y. (2015). Electric Field Screen: Principles and Applications.

[5] Toyoda H., Kusakari S., Matsuda Y., Kakutani K., Xu L., Nonomura T., Takikawa Y. (2019). An Illustrated Manual of Electric Field Screens: Their Structures and Functions.

[6] Jones E., Childers R. (2002). Electric charge and electric field. Physics.

[7] Wegner H.E., Geller E. (2002). Electrical charging generators. McGraw-Hill Encyclopedia of Science and Technology.

[8] Giancoli D.C., Challice J. (2005). Electric charge and electric field. Physics, Principles with Applications.

[9] Matsuda Y., Nonomura T., Kakutani K., Takikawa Y., Kimbara J., Kasaishi Y., Osamura K., Kusakari S.-I., Toyoda H. (2011). A newly devised electric field screen for avoidance and capture of cigarette beetles and vinegar flies. Crop. Prot..

[10] Cross J.A., De Barr A.E. (1987). Dielectrophoresis. Electrostatics: Principles, Problems and Applications.

[11] Kakutani K., Matsuda Y., Haneda K., Sekoguchi D., Nonomura T., Kimbara J., Osamura K., Kusakari S.-I., Toyoda H. (2012). An electric field screen prevents captured insects from escaping by depriving bioelectricity generated through insect movements. J. Electrost..

[12] Kakutani K., Matsuda Y., Haneda K., Nonomura T., Kimbara J., Kusakari S., Osamura K., Toyoda H. (2012). Insects are electrified in an electric field by deprivation of their negative charge. Ann. Appl. Biol..

[13] Helyer N., Brown K., Cattlin N.D., Northcott J. (2004). Pest profiles. A Colour Handbook of Biological Control in Plant Protection.

[14] Kakutani K., Matsuda Y., Nonomura T., Toyoda H., Kimbara J., Osamura K., Kusakari S. (2012). Practical Application of an Electric Field Screen to an Exclusion of Flying Insect Pests and Airborne Fungal Conidia from Greenhouses with a Good Air Penetration. J. Agric. Sci..

[15] Nonomura T., Matsuda Y., Kakutani K., Kimbara J., Osamura K., Kusakari S.-I., Toyoda H. (2012). An electric field strongly deters whiteflies from entering window-open greenhouses in an electrostatic insect exclusion strategy. Eur. J. Plant Pathol..

[16] Matsuda Y., Nonomura T., Kakutani K., Kimbara J., Osamura K., Kusakari S., Toyoda H. Avoidance of an electric field by insects: Fundamental biological phenomenon for an electrostatic pest-exclusion strategy. Proceedings of the Journal of Physics: Conference Series.

[17] Halliday D., Resnick R., Walker J., Johnson S., Ford E. (2005). Electric discharge and electric fields. Fundamentals of Physics.

[18] Kaiser K.L. (2006). Air breakdown. Electrostatic Discharge.

[19] Shimizu K., Matsuda Y., Nonomura T., Ikeda H., Tamura N., Kusakari S., Kimbara J., Toyoda H. (2007). Dual protection of hydroponic tomatoes from rhizosphere pathogens Ralstonia solanacearum and Fusarium oxysporum f.sp. radicis-lycopersici and airborne conidia of Oidium neolycopersici with an ozone-generative electrostatic spore precipitator. Plant Pathol..

[20] Nonomura T., Matsuda Y., Kakutani K., Takikawa Y., Toyoda H. (2008). Physical control of powdery mildew (Oidium neolycopersici) on tomato leaves by exposure to corona discharge. Can. J. Plant Pathol..

[21] Matsuda Y., Takikawa Y., Nonomura T., Kakutani K., Okada K., Shibao M., Kusakari S., Miyama K., Toyoda H. (2018). Selective electrostatic eradication of Sitopholus oryzae nesting in stored rice. J. Food Technol. Pres..

[22] Hill D.S. (1990). Pests of Stored Products and Their Control.

[23] Takikawa Y., Matsuda Y., Kakutani K., Nonomura T., Kusakari S.-I., Okada K., Kimbara J., Osamura K., Toyoda H. (2015). Electrostatic Insect Sweeper for Eliminating Whiteflies Colonizing Host Plants: A Complementary Pest Control Device in An Electric Field Screen-Guarded Greenhouse. Insects.

[24] Takikawa Y., Matsuda Y., Nonomura T., Kakutani K., Okada K., Shibao M., Kusakari S., Toyoda H. (2017). Elimination of whiteflies colonizing greenhouse tomato plants using an electrostatic flying insect catcher. Int. J. Curr. Adv. Res..

[25] Matsuda Y., Kakutani K., Nonomura T., Takikawa Y., Okada K., Shibao M., Miyama K., Yokoo S., Kusakari S.-I., Toyoda H. (2018). A Simple Electrostatic Device for Eliminating Tobacco Sidestream Smoke to Prevent Passive Smoking. Instruments.

[26] Matsuda Y., Takikawa Y., Kakutani K., Nonomura T., Toyoda H. (2020). Analysis of Pole-Ascending–Descending Action by Insects Subjected to High Voltage Electric Fields. Insects.

[27] Newland P., Hunt E., Sharkh S.M., Hama N., Takahata M., Jackson C.W. (2008). Static electric field detection and behavioural avoidance in cockroaches. J. Exp. Biol..

